# Incidence and correlates of mild cognitive impairment, Alzheimer's disease, and related dementias among post‐9/11 Veterans: Findings from the Millennium Cohort Study

**DOI:** 10.1002/alz70860_098360

**Published:** 2025-12-23

**Authors:** Neika Sharifian, Yunnuo Zhu, Felicia R Carey, Wisam Barkho, Elaine Hu, Mary Jo Pugh, Edward J Boyko, Rudolph P. Rull

**Affiliations:** ^1^ Naval Health Research Center, San Diego, CA, USA; ^2^ Leidos Inc, San Diego, CA, USA; ^3^ Seattle Epidemiologic Research and Information Center, Seattle, WA, USA; ^4^ University of Utah, Salt Lake City, UT, USA; ^5^ Informatics, Decision‐Enhancement and Analytic Sciences Center of Innovation, Salt Lake City, UT, USA; ^6^ University of Washington, Seattle, WA, USA

## Abstract

**Background:**

With increases in population aging and life expectancy, the burden of mild cognitive impairment (MCI), Alzheimer's disease, and related dementias (ADRDs) is projected to increase. Veterans, in particular, experience the same risk factors for MCI and ADRDs as the general U.S. population (e.g., aging, genetic risk) as well as service‐specific risk factors (e.g., traumatic brain injury, posttraumatic stress disorder [PTSD], occupational exposures) that may put them at greater risk.

**Method:**

The current study aimed to examine the incidence and correlates of MCI and ADRDs in post‐9/11 veterans utilizing over 20 years of longitudinal data from the Millennium Cohort Study (*n* = 146,670). MCI and ADRD cases were ascertained through ICD‐9 and 10 codes obtained through Veterans Health Administration and Military Health System Data Repository electronic health records between 2001‐2024. Age‐ and sex‐adjusted rates were reported per 100,000 person‐years calculated from the date of birth and censored at the date of diagnosis, date of death, or end of the observation period (August 15^th^, 2024), whichever came first. Age‐ and sex‐adjusted rates by characteristic subgroups were estimated from direct standardization methods and standardized to the total military population in 2010.

**Result:**

During the study period, 1,759 veterans were diagnosed with MCI (25.82 cases per 100,000 person‐years, 95% CI=24.25, 27.38) and 661 veterans were diagnosed with ADRD (6.86 cases per 100,00 person‐years, 95% CI=6.17, 7.54). A majority of ADRDs did not have an identified etiology (e.g., unspecified dementias). Among those who did, Alzheimer's disease and vascular dementia were the most prevalent diagnoses within the cohort. Military characteristics, such as serving in the Army and in occupations with high risk for low‐level blast, were associated with higher age‐ and sex‐adjusted rates of MCI and ADRD diagnoses. Mental (e.g., PTSD, depression) and physical health (e.g., hearing loss, smoking) factors were also associated with higher rates of MCI and ADRDs.

**Conclusion:**

Findings highlight potential unique military risk factors for the development of cognitive impairment and dementia. These findings may help to inform future policy and develop interventions aimed at reducing long‐term cognitive health consequences that may be associated with military service.

